# Fatal Microangiopathic Hemolytic Anemia Due to Sézary Syndrome

**DOI:** 10.7759/cureus.15482

**Published:** 2021-06-06

**Authors:** Jake C Robertson, Mustufa A Jafry, Lori Soma, Andrei Shustov, Michi M Shinohara

**Affiliations:** 1 School of Medicine, University of Washington, Seattle, USA; 2 Division of Hematopathology, Department of Pathology, University of Washington, Seattle, USA; 3 Division of Hematology, Department of Medicine, University of Washington, Seattle, USA; 4 Division of Dermatology, Department of Medicine, University of Washington, Seattle, USA

**Keywords:** sézary syndrome, lymphoma, anemia, thrombocytopenia, hemolysis

## Abstract

Sézary syndrome (SS) is a form of cutaneous T-cell lymphoma (CTCL), demonstrating leukemic involvement of malignant T-cells. Known systemic sequelae of SS include hemophagocytic syndrome-induced anemia, normocytic anemia secondary to bone marrow infiltration, and pancytopenia. We report a patient with SS, initially demonstrating widespread morbilliform eruption, who presented with malignancy-related microangiopathic hemolytic anemia (MAHA). Our findings represent a novel presentation of SS that will inform the differential diagnosis and treatment of future SS patients presenting with anemia and thrombocytopenia.

## Introduction

Sézary syndrome (SS) is a subtype of cutaneous T-cell lymphoma (CTCL) primarily originating from matured T-lymphocytes that are present in the skin and blood [1]. SS affects older patients primarily over the age of 60, is more common in males, and has an incidence of about 3:1,000,000 in the US [2]. While anemia and thrombocytopenia have previously been reported in patients with SS secondary to bone marrow infiltration, autoimmune hemolysis, or treatment with particular antineoplastic agents, there have not been previous reports of a microangiopathic hemolytic anemia (MAHA) secondary to primary SS [3].

## Case presentation

A 77-year-old female was diagnosed with SS on the basis of a pruritic, subtle but widespread morbilliform eruption. Skin biopsy revealed interface and focally lichenoid lymphocytic infiltrate with some mildly atypical cells and minimal epidermotropism. Peripheral blood flow cytometry revealed an atypical T-cell population of CD4+/CD5+ T-cells, with a CD4/CD8 ratio of 10.5, and loss of CD7. She was diagnosed with SS with a Sézary count of 6,600 cells/µL. Her symptoms were thereafter well controlled with topical triamcinolone ointment 0.1% used once daily, and the patient opted for no systemic therapy for her SS. She was followed closely with physical exams and laboratory studies and noted over the next several years to have a slow increase in circulating Sézary count and elevation of the CD4/CD8 ratio in the blood. A concomitant decrease in the overall percent of natural killer (NK) cells and mild thrombocytopenia without any associated bleeding or purpura was also seen. 

**Figure 1:**
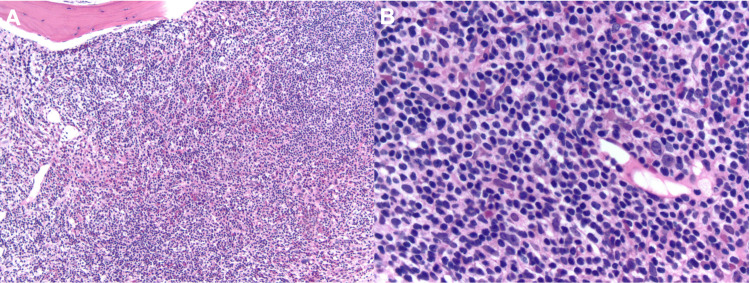
Bone marrow biopsy shows infiltration with 70% Sézary cells by morphology. (A) x10 magnification and (B) x40 magnification.

**Figure 2:**
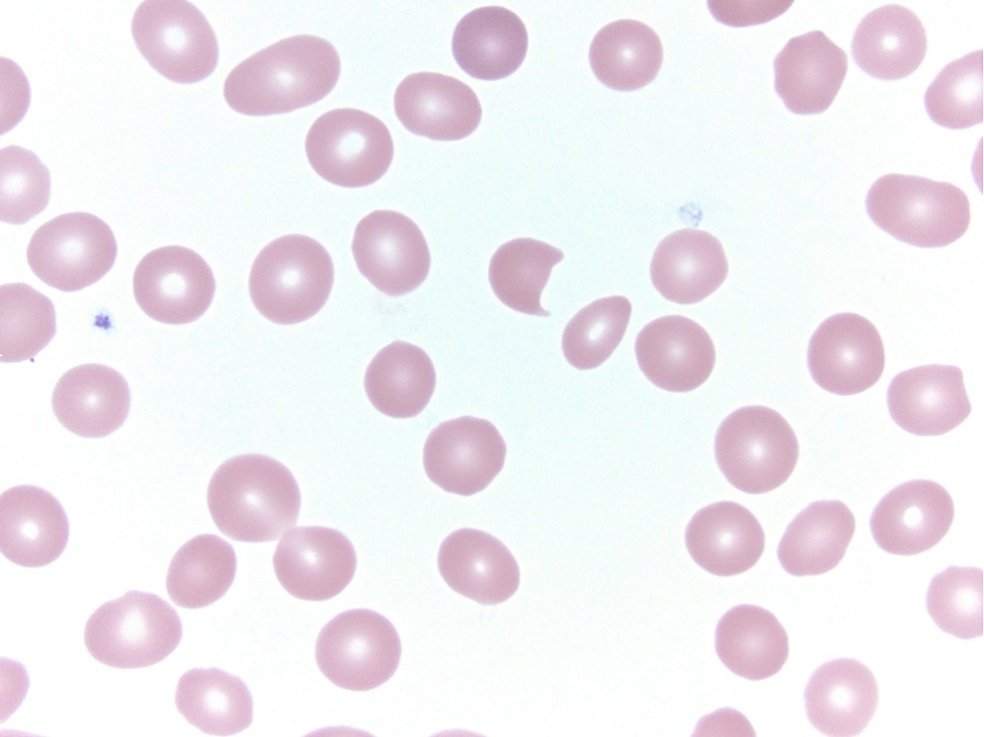
Peripheral blood smear shows the presence of schistocytes, indicative of a microangiopathic-hemolytic process.

Three years after her initial presentation, the patient presented with fatigue, shortness of breath, and lower extremity swelling. Workup at that point revealed a marked anemia with a hemoglobin of 4.1 g/dL and a hematocrit of 12%, a total white cell count of 24.1 thousand cells/µL, a mean corpuscular volume (MCV) of 105 fl, a red cell distribution width (RDW) at 21.8, a platelet count of 138,000 cells/µL, an absolute reticulocyte count of 71.0/µL, a lactate dehydrogenase (LDH) at 262 units/L, a total bilirubin of 2.8 µmol/L, and a haptoglobin at 35 mg/dL. Direct antiglobulin test (DAT) was negative. A bone marrow biopsy (Figures 1A, 1B) at the time revealed marked involvement by her T-cell neoplasm with over 70% Sézary cells by morphology; concurrent flow cytometry of the bone marrow confirmed 61.9% T-cells with slightly decreased expression of CD2, uniformly positive expression of CD4, slightly increased expression of CD5, variably decreased expression of CD7, and loss of CD26. Peripheral blood smear displayed schistocytes (Figure 2). With the patient’s elevated LDH, decreased haptoglobin, schistocytes, negative Coombs, near upper limit reticulocyte count, and severe anemia, she was diagnosed with a non-autoimmune, MAHA in the setting of SS.

The patient required multiple blood transfusions for stabilization of her hematocrit. She was started on alemtuzumab 10 mg by intravenous infusion three times a week soon after to treat her SS. Within two months, her hemoglobin and platelet levels had stabilized without further transfusion, and she remained stable for six months. The patient then developed recurrent MAHA that ultimately failed to respond to further therapy. She was transitioned to comfort care and died from her disease four years after the initial presentation.

## Discussion

SS has been associated with hemophagocytic syndrome-induced anemia, normocytic anemia secondary to bone marrow infiltration, and pancytopenia [4-6]. Thrombotic thrombocytopenic purpura-hemolytic uremic syndrome (TTP-HUS) has been observed as dose-dependent toxicity of pentostatin treatment for SS [7]. However, no single, common form of anemia has been found to be present in all SS patients.

Other diagnoses considered in the differential diagnosis for our patient included anemia secondary to decreased production from infiltration of the bone marrow. While the bone marrow biopsy did reveal advanced disease, the patient had an elevated reticulocyte count, demonstrating capacity for bone marrow production. Autoimmune hemolytic anemia was also a consideration and has been previously reported in patients with SS [8]. In this case, the patient’s DAT was negative. Lastly, the presence of schistocytes on the peripheral blood smear made MAHA the most likely diagnosis. Hemolytic uremic syndrome (HUS) and thrombotic thrombocytopenic purpura (TTP) were given consideration in this context, but with normal renal function and negative fever or neurologic symptoms, both diagnoses were essentially excluded.

Malignancy-associated MAHA has been previously reported [9-11]. The most common malignancies associated include colon, gastric, prostate, lung, and carcinoma of unknown primary (CUP). In many of these cases, MAHA was one of the first presentations of malignancy, and extensive workup for TTP, HUS, and disseminated intravascular coagulation (DIC) were all negative. Potential pathophysiological mechanisms for the association of MAHA and malignancy include arteriolar or capillary swelling secondary to endothelial damage of the marrow vasculature from the invasion of the malignancy, leading to the production of large Von Willebrand Factor (VWF) multimers and subsequently creating a microangiopathic hemolytic process similar to TTP [9,10,12]. Similarly, it has been proposed that tumor emboli or intraluminal fibrin thrombi secondary to hypercoagulation may lead to direct blood vessel damage, creating an environment for RBC fragmentation [9,10,12,13]. In the case of gastric cancers, the production of endothelium-damaging mucin has been proposed as a potential mechanism for direct blood vessel damage [14].

The primary treatment for malignancy-associated MAHA is the treatment of the underlying malignancy [9,10]. In many cases, control of the primary malignancy has subsequently controlled the hemolytic anemia and stabilized blood counts [15]. Treatments utilized for TTP-HUS, such as recombinant ADAMTS 13 and plasma exchange, have been shown to demonstrate efficacy in treating malignancy-associated MAHA [16]. The average survival of malignancy-related MAHA is four months for patients receiving anti-cancer therapy (including chemotherapy or cancer surgery), and 0.5 months for those not receiving anti-cancer therapy, with associated subgroup variation by type of malignancy [15].

## Conclusions

In conclusion, we present a case of a patient with SS presenting with malignancy-associated MAHA. It is important for treating hematologists/oncologists and dermatologists who encounter patients with anemia and thrombocytopenia in the setting of SS to not only rule out HUS, TTP, and anemia secondary to direct infiltration of the bone marrow, but to also recognize that MAHA can occur in this setting. Treatment of malignancy-associated MAHA primarily includes control of the underlying malignancy; further investigation is required to determine efficacious treatment modalities specific to malignancy-associated MAHA.
